# Using hybrid pre-trained models for breast cancer detection

**DOI:** 10.1371/journal.pone.0296912

**Published:** 2024-01-22

**Authors:** Sameh Zarif, Hatem Abdulkader, Ibrahim Elaraby, Abdullah Alharbi, Wail S. Elkilani, Paweł Pławiak

**Affiliations:** 1 Department of Information Technology, Faculty of Computers and Information, Menoufia University, Shebin El-kom, Menoufia, Egypt; 2 Artificial Intelligence Department, Faculty of Artificial Intelligence, Egyptian Russian University, Cairo, Egypt; 3 Department of Information Systems, Faculty of Computers and Information, Menoufia University, Shebin El-kom, Menoufia, Egypt; 4 Department of Information Systems Management, Higher Institute of Qualitative Studies, Cairo, Egypt; 5 Department of Computer Science, Community College, King Saud University, Riyadh, Saudi Arabia; 6 College of Applied Computer Science, King Saud University, Riyadh, Saudi Arabia; 7 Department of Computer Science, Faculty of Computer Science and Telecommunications, Cracow University of Technology, Krakow, Poland; BOU: Bangladesh Open University, BANGLADESH

## Abstract

Breast cancer is a prevalent and life-threatening disease that affects women globally. Early detection and access to top-notch treatment are crucial in preventing fatalities from this condition. However, manual breast histopathology image analysis is time-consuming and prone to errors. This study proposed a hybrid deep learning model (CNN+EfficientNetV2B3). The proposed approach utilizes convolutional neural networks (CNNs) for the identification of positive invasive ductal carcinoma (IDC) and negative (non-IDC) tissue using whole slide images (WSIs), which use pre-trained models to classify breast cancer in images, supporting pathologists in making more accurate diagnoses. The proposed model demonstrates outstanding performance with an accuracy of 96.3%, precision of 93.4%, recall of 86.4%, F1-score of 89.7%, Matthew’s correlation coefficient (MCC) of 87.6%, the Area Under the Curve (AUC) of a Receiver Operating Characteristic (ROC) curve of 97.5%, and the Area Under the Curve of the Precision-Recall Curve (AUPRC) of 96.8%, which outperforms the accuracy achieved by other models. The proposed model was also tested against MobileNet+DenseNet121, MobileNetV2+EfficientNetV2B0, and other deep learning models, proving more powerful than contemporary machine learning and deep learning approaches.

## 1. Introduction

Breast cancer represents the most commonly diagnosed form of cancer and ranks as the second leading cause of cancer-related deaths on a global scale. [1]. Currently, there are more than 3.8 million women who suffer from breast cancer diagnoses. Breast cancer is the most common type diagnosed among women in the United States, indicating its high prevalence [2]. According to the American Cancer Society in the United States, in 2023, increasing by about 0.5% per year, there will be approximately 297,790 diagnoses of invasive breast cancer and 43,700 deaths due to breast cancer [3]. The accurate diagnosis of breast cancer is often hindered by the intricate characteristics of microcalcification and masses, leading to challenges faced by radiologists [4]. Early detection and proper diagnosis are crucial for improving treatment outcomes and increasing survival rates for those affected by this disease.

An innovative way to obtain additional insights into breast cancer and enhance the accuracy of a physician’s detection A computer-aided detection and diagnosis (CAD) system is used to analyze medical images, improve significantly cost-effective screening performance, mostly because of the low specificity, and assist in detecting and diagnosing medical conditions. These systems utilize algorithms to extract relevant features from the images for further analysis, leading to accurate decisions. The computer output is more precise compared to radiologists’ diagnoses due to differences in diagnoses that can be significant [5, 6]. The FDA recognized computer-aided detection as a medical device in 1998 [7].

Thus, the significance of this research lies in offering a solution for breast cancer diagnosis through artificial intelligence (AI) and deep learning techniques. Nevertheless, it is imperative to conduct a thorough critical assessment of the efficacy of artificial intelligence (AI) prior to its implementation as a means for the autonomous interpretation of mammograms [8].

AI has been increasingly integrated into various industries, including the healthcare sector. AI’s significance in the medical industry cannot be overstated due to its potential to revolutionize how medical professionals diagnose and treat patients [9]. Its usage is becoming more prevalent in the medical field, and it has the potential to serve as a valuable supplement to traditional clinical practices, especially for breast cancer detection as early as possible, where AI algorithms can analyze images and support accurate diagnoses. Additionally, with the integration of AI, radiologists commonly utilize various imaging technologies used in medicine, including mammography, magnetic resonance imaging, ultrasound, computed tomography imaging, and others, to diagnose breast cancer [10, 11]. The rise of deep learning has been a major contributor to the AI revolution, making it possible to create predictive models using medical imaging data. However, despite the limitations, deep learning in digital histopathology analysis has shown promise for improving accuracy and reducing the burden on human pathologists [12]. Therefore, the use of artificial intelligence and deep learning methodologies in the analysis of digital histopathology images is an area of ongoing research and development with the potential to revolutionize the field of breast cancer diagnosis [13]. To enhance the accuracy of breast cancer screening. By incorporating deep learning techniques, this study aims to overcome the limitations and challenges faced by traditional AI-based methods and provide a more efficient and effective solution for breast cancer diagnosis [14]. With the help of deep learning, this paper seeks to transform how breast cancer screening is performed and ultimately improve patient outcomes.

However, by utilizing deep learning models for detecting breast cancer, we will compare the output accuracy of several pre-trained models individually to obtain the highest accuracy. This study focuses on enhancing the performance of breast cancer detection by integrating multiple pre-trained models, aiming to improve classification accuracy and make BC-IDC tissue detection more efficient. This research offers a unique contribution to the field through the following:

- Propose a new integration technique for detecting breast cancer that integrates multiple CNN models. When compared to other ways of learning, the suggested method was respectable for detecting BC-IDC tissues in breast cancer whole slide images (WSI).
- Evaluation and comparison of the proposed model with other state-of-the-art deep learning models (MobileNet+DenseNet121, MobileNetV2+EfficientNetV2B0, etc.) to show its better or higher performance in regards to accuracy, precision, recall, F1-score, MCC, (ROC-AUC), and (AUPRC).
- We are utilizing the knowledge transferred from multiple pre-trained models to enhance the accuracy and efficiency of detecting and classifying IDC tissues in the breast cancer histology images from Kaggle.

The proposed hybrid deep learning model is expected to assist pathologists in making more accurate diagnoses of breast cancer, thereby contributing to advancing breast cancer screening and diagnosis.

In this work, a novel CNN+EfficientNetV2B3 hybrid model is proposed for breast cancer classification, and the results indicate its superior performance over the existing models.

The present paper is organized in the following manner: Section 2 provides the literature review on deep learning detection and classification techniques for breast cancer diagnosis. The proposed method used in this study is outlined in Section 3. The study’s findings and data analysis are presented in Section 4, while Section 5 includes a comparative analysis. The paper concludes with a summary and recommendations for future work in Section 6.

## 2. Related work

This section reviews prior research on breast cancer detection utilizing CNNs as feature extractors [15]. Some studies have utilized CNNs to extract features, which are then fed into a classifier to make a final prediction on the label of an image [16, 17]. The basic idea behind a CNN is to extract high-level features from an image using a series of convolutional, pooling, and fully connected layers. These layers work together to identify and extract the most important information from the image, allowing for more accurate classification and detection [18]. This section summarizes the recent developments and advancements in breast cancer detection, particularly in using standard CNN architecture and methods to enhance its performance. The focus is on presenting existing works related to the method proposed in this study.

Barsha et al. [19] presented a model for detecting BC-IDC using DenseNet-121 and DenseNet-169 with test time augmentation (TTA). The accuracy achieved by this model was 92.70%, and utilizing pre-trained models, including Dense-Net-121, DenseNet-201, ResNet-101v2, and ResNet-50, an ensemble model was proposed for the classification of BC-IDC. The model is inferred from two cohorts of validation. Concerning the patch level classification, the models achieved 69.31%, 75.07%, and 61.85% accuracy, and 60.50% in the first model and 62.44%, 79.14%, 76.62%, and 71.05% in the second model for 4x, 10x, 20x, and 40x magnified images, respectively. Using a distinct IDC dataset, the same architecture is further validated, achieving an overall accuracy of 90.07%. Wang et al. [20] demonstrated the effectiveness of combining CNN and GRU in detecting BC-IDC and using oversampling techniques and data augmentation for model performance improvements with an accuracy of 86.21%. Choudhary et al. [21] proposed a novel approach for histopathological image classification using a deep CNN-based transfer learning method with structured filter pruning. The approach aimed to reduce the runtime resource requirements of deep learning models that have been trained while maintaining or even improving their accuracy. This method begins by removing less significant filters from convolutional layers, followed by training the remaining experiments on the histopathological image dataset using different models along with VGG19, ResNet34, and ResNet50, three prominent pre-trained CNNs; with the VGG19 model, the VGG19 model achieved accuracy of 91.25%, the ResNet34 model achieved 91.80% accuracy, and the ResNet50 model achieved accuracy of 92.07%. Zeng et al. [22] The authors experimented with a machine learning (ML) model using Google Cloud AutoML Vision instead of a manually designed neural network. The model was built using histopathology images as the original dataset, and positive images were rotated to balance the number of IDC negative and IDC positive images. During the model evaluation, this method achieved an average accuracy of 91.6%. Celik et al. [23] developed a model for detecting invasive ductal carcinoma (IDC) using the BreakHis breast cancer dataset and utilizing ResNet-50 and Dense-Net-161 pre-trained deep learning techniques. The aim was to detect IDC through the use of these pre-trained models. They obtained an accuracy of 91.57% via DenseNet-161. Abdolahi et al. [24] presented two deep-learning techniques for classifying IDC in histopathology images. A simple CNN (named the baseline model) was trained in the first approach. The second method utilized the VGG-16 model for feature extraction, fine-tuning, and classification of breast disease images. The baseline model accuracy for the automatic classification of IDC terms was 85%. Zhang et al. [25] proposed an approach for automatically detecting BC-IDC-based MSRCNN-SVM. After cutting the whole slide image (WSI) into patches based on the coordinates and preprocessing the data through enhancement and normalization, they are input into MSRCNN to extract features. The features are fed into SVM for classification, and finally, in the WSI, the IDC (-) and IDC (+) regions are drawn. Automated identification of invasive ductal carcinoma (IDC) is realized by small-slice classification. In the 5-fold cross-validation, the average accuracy for the MSRCNN-SVM model was 87.45%. Othman et al. [13] propose a deep-learning model using CNNs to detect liver tumors from CT scans. These models use hybrid models based on pre-trained DeeplapV3 with ResNet-50 and VGG-16, ResNet-50 V2, and U-Net with LSTM for liver tumor detection and segmentation. The experimental results showed that the first method obtained high accuracy. Ibrahim et al. [26] presented a new combination of DL models based on CNNs for liver tumor detection using CT scans. The researchers utilized DeeplapV3 + ResNet-50 and VGG-16 + ResNet-50 V2 + U-Net++; these methods were highly accurate in the diagnosis of liver tumors. Sharmin et al. [27] In this study, a novel hybrid model is proposed for the reliable detection of breast cancer that integrates deep learning (DL) and machine learning (ML) techniques. This model utilizes the capabilities of the deep learning-based pre-trained ResNet50V2 transfer learning model to extract intricate patterns and representations from invasive ductal carcinoma (IDC) pictures effectively. The usefulness of this strategy was established by experiments on a comprehensive dataset of IDC, resulting in high accuracy. The findings yielded a significantly enhanced accuracy of 95%.

However, most of these studies are not robust and obtain low performance, such as Wang et al. [20], Abdolahi et al. [24], and Zhang et al. [25], which demand analysis approaches to improve the model’s robustness. Barsha et al. [19], Zeng et al. [22], and Celik et al. [23] received low classification results. In addition, some works are computationally complex, such as Choudhary et al. [21], Othman et al. [13], and Ibrahim et al. [26], trained on small data emphasizes the requirement for a stronger and more all-encompassing technique for breast cancer diagnosis using deep learning methods.

The proposed method in this study aims to address the limitations of previous studies by introducing a combination of several CNN models used together for the first time in breast cancer classification. The aim is to achieve improved performance and higher accuracy rates than previous methods. Additionally, the method is designed to be more robust and efficient in terms of calculation costs, reducing computational demands while still providing accurate results.

## 3. Methodology

**Fig 1:**
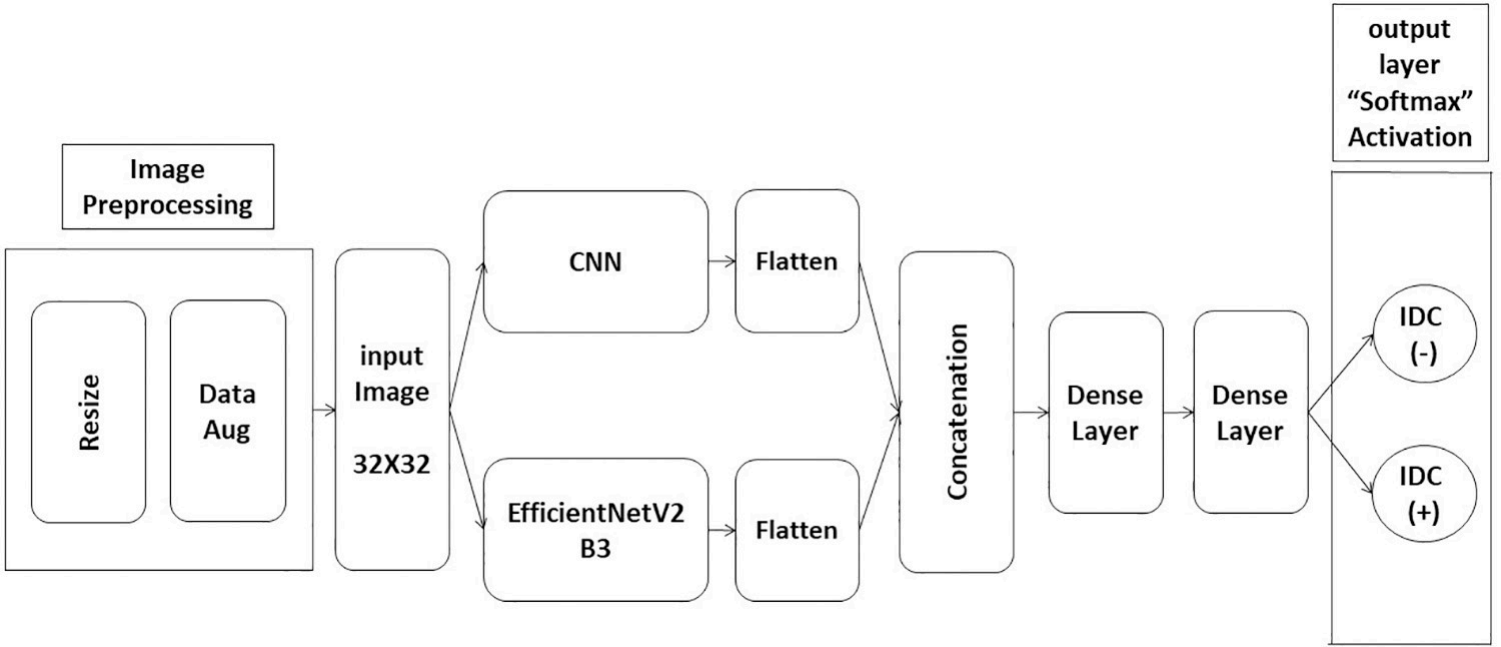
The components of the proposed model.

We focus on the dataset and proposed method used for breast cancer classification. The dataset used for the model is discussed in detail to provide insights into the model’s training and testing data. Additionally, the method suggested is presented comprehensively, including an overview of the integration of multiple CNN models and the approach used to overcome previous limitations. A graphical representation of the proposed method is also illustrated in Fig 1.

In Fig 1 above, the data preprocessing is described in Section 3.2. The concatenation after flattening in hybrid models is utilized to combine the outputs from the two models or branches, then introduced to a fully connected network consisting of two dense layers and an output layer with softmax activation.

### 3.1. Breast histopathology images dataset

**Fig 2:**
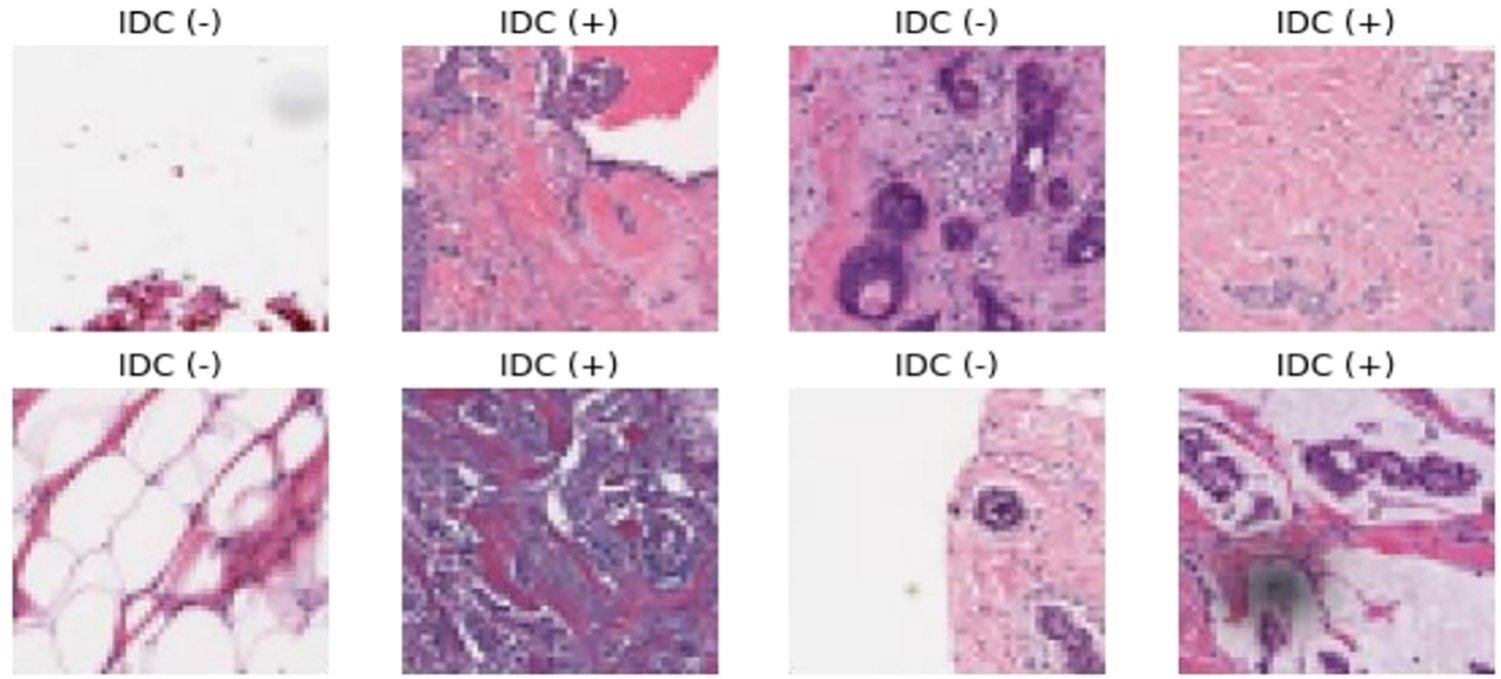
The class labeling of IDC negative and IDC positive in the IDC dataset.

The dataset utilized in this study was acquired from the Kaggle open-access database, a well-known platform for machine learning and data science competitions. The dataset was publicly accessible, allowing easy access for this research. The dataset details, including the number of samples and types of features, are discussed in [28]. The dataset contained 162 whole-mount slide images with IDC tissue regions in whole slide images (WSI) of breast cancer gathered from the Pennsylvania University Hospital and the New Jersey Cancer Institute. 277,524 images with a size of 50 x 50 pixel RGB. The dataset included in this study consisted of 162 breast histopathology samples that were stained with H&E. The images were captured at 40 x. The file name for each patch follows a certain format: u_xX_yY_classC.png. For example, a file name could be 10268_idx5_x1601_y1251_class1.png. In this context, the patient ID (10268_idx5) is denoted as u, X represents the x-coordinate of the patch’s cropping location, the y-coordinate of the patch’s cropping location is represented by Y, and the class is indicated by C, where 0 signifies non-IDC and 1 signifies IDC. The dataset is divided into 198,738 images that are negative (benign) and 78,786 images that are positive (malignant), which is highly imbalanced. Sample images are given in Fig 2.

This study randomly selected 30% of the images for testing, and 70% represented the training set. The network’s training will be carried out with the optimization of various parameters, such as the optimizers, epochs, batch size, etc., through an iterative process that involves a customized CNN and transfer learning using popular computer vision networks will ensure that the model is fine-tuned and achieves optimal performance. The model’s performance is evaluated by calculating its loss and accuracy on the training and testing datasets. The results are then visualized using graphs, confusion matrices, and various performance metrics, such as accuracy, MCC, ROC-AUC, and AUPRC, to evaluate the model’s performance.

### 3.2. Data preprocessing

**Fig 3:**
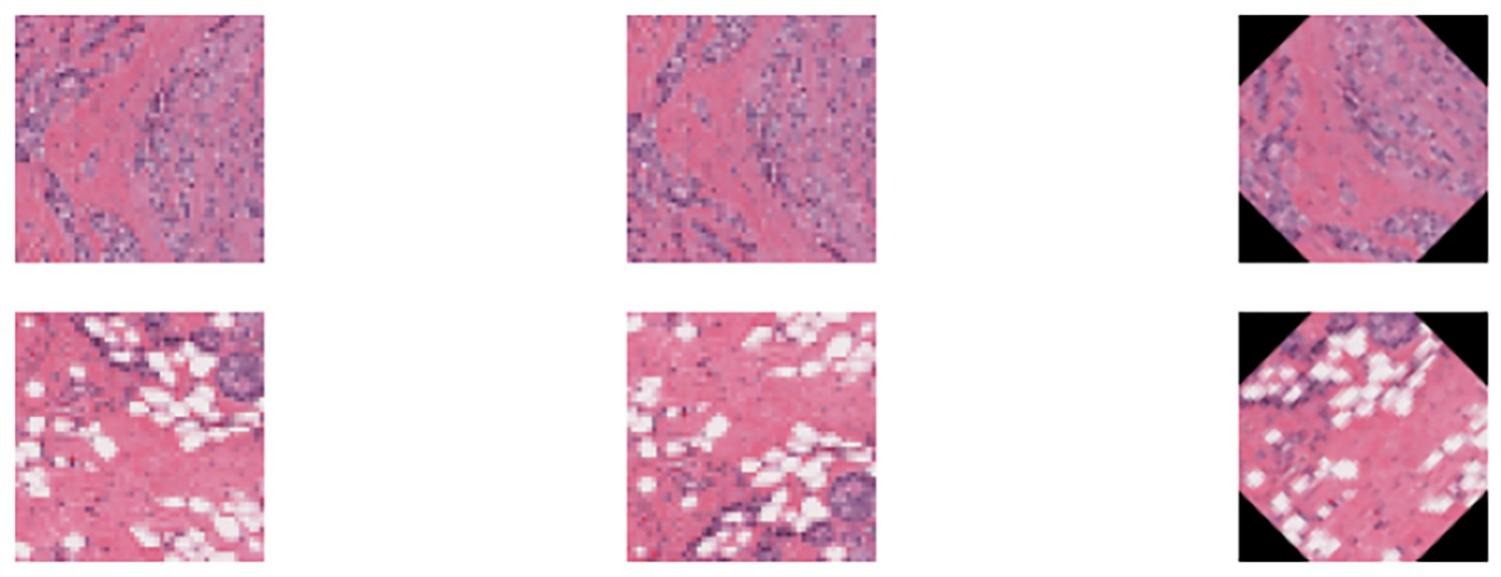
Examples of the Input IDC images after applying augmentation.

Data preprocessing is a critical stage in developing an accurate and efficient deep-learning model. In our study, we performed two main preprocessing steps on the BC-IDC dataset. First, we resized all images from their original size of 50x50 pixels to 32x32 pixels in order to speed up the training process without compromising the quality of the images, especially with limited computing resources, which increases the saturation of the random access memory (RAM). Consequently, it became necessary to restart the training process from its initial stage. To mitigate this issue, we opted to decrease the dimensions of the images. The method chosen for resizing images in OpenCV Lib is a bilinear interpolation; it takes a weighted average of four nearest pixels around the target location to calculate the new pixel value. Second, we applied image augmentation to all positive images by two methods: flipping images horizontally and then rotating images by 45 degrees. This technique helped address the class imbalance between positive and negative cases. By flipping and rotating the positive images, we increased the number of positive samples in the dataset and improved the accuracy and reliability of the model’s predictions. Together, these preprocessing steps played a critical role in improving the performance of the proposed hybrid models for breast cancer classification. Examples of the images after using augmentation techniques are shown in Fig 3.

### 3.3. Cross-entropy loss function

The cross-entropy function is widely used in deep learning, calculating the difference between predicted probability and actual class, which can be 0 and 1, and optimizing classification models for the binary cross-entropy formula given below:

BinaryCrossEntropy(y,yˆ)=−(ylog(yˆ)+(1−y)log(1−yˆ)). (1)

Where N is the number of examples, y, yˆ Є {0, 1} N, Y is the actual label, and yˆ is the predicted value, and for multiclass classification tasks, the categorical cross-entropy is defined as:

$$Categorical\;Cross\;Entropy(y,p)=-\frac{1}{N}\sum_{i=1}^{N}\sum_{c=1}^{C}y_{i,c}.\log\left(p_{i,c}\right)$$ (2)

Here, yi,c uses a one-hot encoding of the actual class, pi,c refers to a matrix of predicted values for every class, and c, i refers to iterating over all classes and pixels. In this study, we employed binary cross-entropy, the most prevalent loss function for binary classification tasks.

### 3.4. Proposed BC-IDC detection models

This section proposes an efficient breast cancer classification approach that integrates several pre-trained CNN models. Our method leverages the strengths of different CNN models to improve accuracy and robustness while reducing calculation costs. The proposed models are explained in detail.

The proposed combinations are as follows:

The proposed model integrates three different pre-trained models: (1) a combination of Customized CNN and EfficientNetV2B3 [29], (2) a combination of MobileNet [30] and Densenet121 [31], and (3) a combination of MobileNetV2 [32] and EfficientNetV2_b0 [33]. These models were chosen to leverage their pre-existing knowledge and weights that were previously trained for IDC detection.

The combination of each two models’ backbones by using the conv part, then adding a flattening layer to each model, and then the concatenation layer combines both outputs. The concatenation layer in deep learning merges or combines the outputs of multiple layers or branches. It’s used to provide a more comprehensive set of features by stacking outputs along a particular axis, enhancing the model’s ability to learn from various sources of information simultaneously.

Hybrid architectures often demonstrate increased success in various machine learning tasks due to their ability to capture diverse features and representations. By combining multiple models or architectures, they can collectively leverage the strengths of each component.

The hybrid architecture effectively benefits from both, improving the capacity to detect and learn various types of features, including but not limited to edges, textures, object parts, and abstract representations. This collective feature extraction enhances the overall performance and the system’s ability to discriminate between different classes or entities within the data.

#### 3.4.1. First model (CNN + EfficientNetV2B3)

The EfficientNetV2 is a new family of CNNs with better parameter efficiency and faster training speed than previous architectures. It produced the best results and the highest efficiency in classifying several classes of the ImageNet dataset. For example, the EfficientNetV2B3 architecture, which admits input images with 32x32 pixels and has 14.5M parameters with 95.8% accuracy on ImageNet [34, 35], is a suitable model for medical image classification.

The proposed hybrid model combines the CNN model with the EfficientNetV2B3 architecture. The CNN model consists of 5 blocks of convolutional layers, where each block includes two convolutional layers, followed by batch normalization and ReLU activation functions. The first block has 32 filters, while the subsequent blocks have 64, 128, and 256 filters, respectively. Additionally, dropout layers and max pooling follow each block. The input shape of the CNN model is (x, y, 3), where x and y are the width and height of the resized images, respectively. The input to the CNN branch is also resized from 50 x 50 pixels to 32 x 32 pixels.

**Fig 4:**
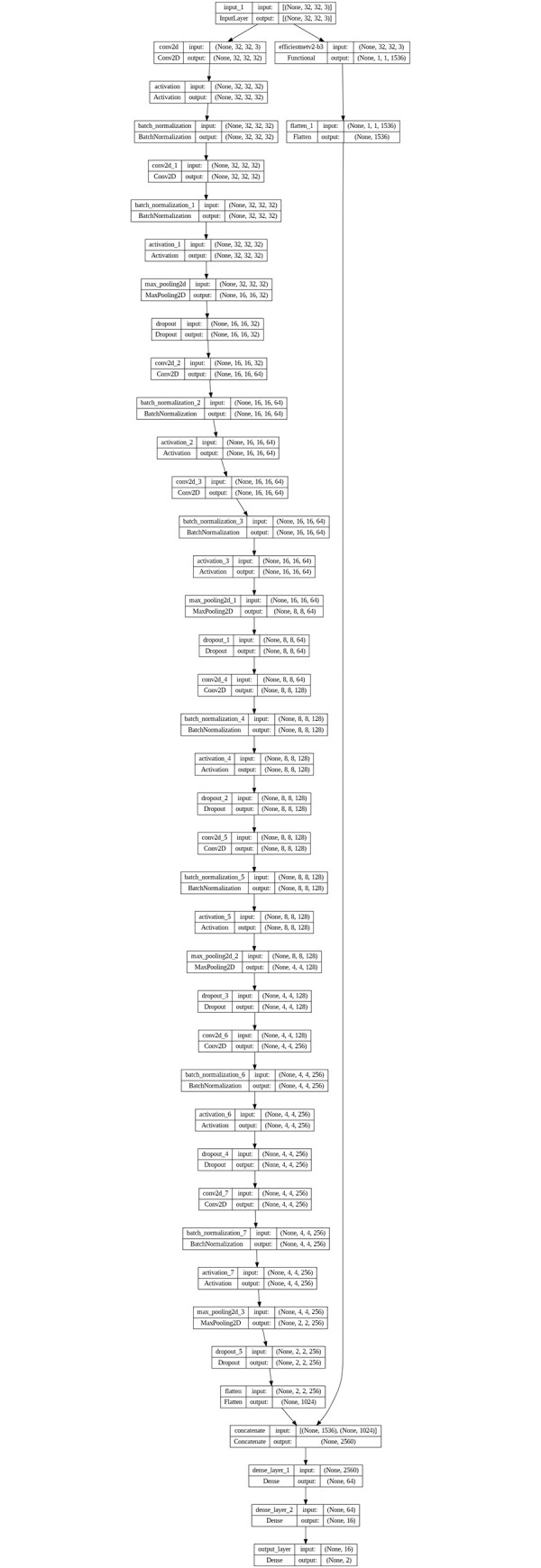
The architecture of the first model.

On the other hand, the EfficientNetV2B3 [29] architecture has 24 layers, including 22 convolutional layers, one fully connected layer, and 1 Softmax layer. The convolutional layers are organized into eight blocks, each consisting of a combination of convolutional layers, squeeze-and-excitation layers, and mobile inverted bottleneck layers. The first convolutional layer has 40 filters, while the subsequent blocks have 48, 96, 136, 232, 384, 1536, and 1536 filters, respectively. The EfficientNetV2B3 architecture also includes global average pooling, dropout, and batch normalization layers, which help to reduce overfitting and improve model performance. The input to the EfficientNetV2B3 branch is also resized from 50 x 50 pixels to 32 x 32 pixels. The model configuration is shown below in Fig 4, and the total number of parameters in this model is 14,271,696.

The output of each branch is concatenated by stacking the two outputs and passed through two dense layers with 64 and 16 units, respectively. The SoftMax activation function is commonly used in binary classification tasks. The model is trained by employing the binary cross-entropy loss function in conjunction with the Adam optimizer.

The CNN+ EfficientNetV2B3 model worked better when one of the advanced pre-trained models, the EfficientNetV2B3 model, was added. The EfficientNetV2B3 model took high-level representations and semantic data from the input images as a feature extractor. By utilizing the pre-trained weights of the EfficientNetV2B3 model, this model was able to benefit from transfer learning and avoid training the entire model from scratch, resulting in faster convergence and enhanced accuracy.

The outputs of the EfficientNetV2B3 model and the CNN model are combined. Next, a series of completely linked, fully connected layers with sigmoid activation functions are applied to this composite representation. Two dense layers with softmax activation comprise the final output layer, representing the binary classification of IDC (+, -).

Finally, the suggested approaches were evaluated using various assessment metrics, such as accuracy, precision, recall curve, F1-score, MCC, AUC, and AUPRC. The performance of this model was extensively assessed by considering various assessment measures. By comparing the CNN+EfficientNetV2B3 model with other breast cancer detection techniques, the CNN+EfficientNetV2B3 model obtained high accuracy and proved its superiority.

The maximum accuracy was reached by combining the CNN with the EfficientNetV2B3 model, data augmentation approaches, powerful optimization algorithms, and thorough assessment metrics.

#### 3.4.2. Second model (MobileNet + DenseNet121)

The proposed hybrid model uses transfer learning to combine the strengths of two pre-trained models, MobileNet [30] and DenseNet121 [31], for more robust breast cancer classification. The internal architecture of the model consists of two branches, with each branch, using a pre-trained model to extract features from the input image.

There are 28 convolutional layers in the MobileNet model, with a depthwise convolution and a pointwise convolution following. This architecture allows for efficient computation and a smaller number of parameters. The input to the MobileNet branch is resized from 50 x 50 pixels to 32 x 32 pixels, which helps reduce the computational complexity and improves the training speed.

The DenseNet121 model, on the other hand, has 121 convolutional layers with dense connections between each layer. This architecture allows for better feature reuse and gradient flow, improving accuracy. The input to the DenseNet121 branch is also resized from 50 x 50 pixels to 32 x 32 pixels.

**Fig 5:**
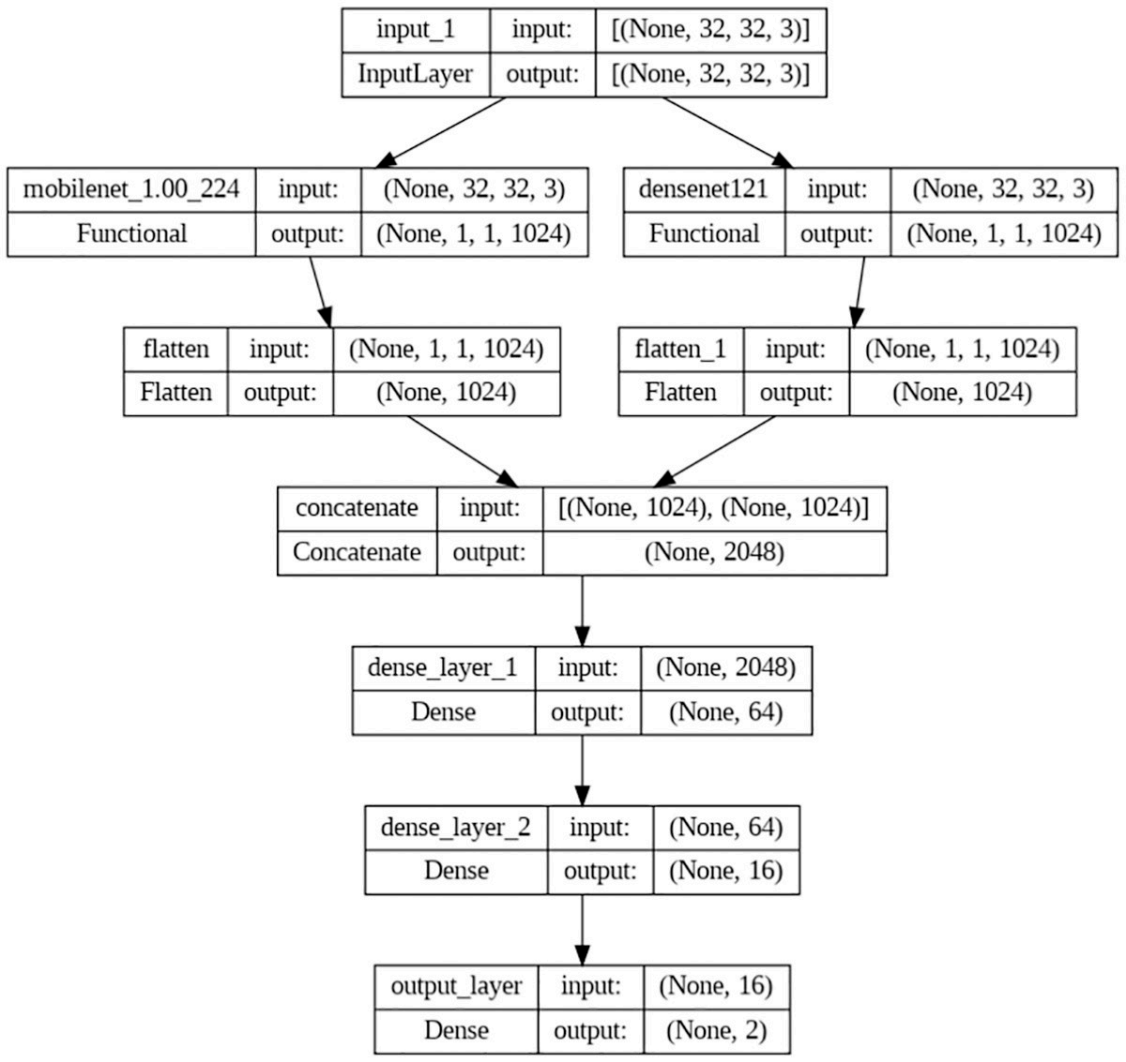
The architecture of the second model.

Combining MobileNet and DenseNet121 in a hybrid model can leverage both models’ strengths and improve the model’s overall performance. The depthwise separable convolutions of MobileNet can help reduce the computational cost and memory footprint, while the dense connectivity of DenseNet121 can help improve feature propagation and reuse. The output from both branches can be concatenated and fed into dense layers for binary classification using SoftMax activation. Overall, this hybrid model can provide a more robust and accurate solution for breast cancer classification. The model configuration is shown below in Fig 5, with trainable parameters of 10,398,578.

Each branch’s output from the last convolutional layer is concatenated together and sent through two dense layers with 64 and 16 units, respectively, along with the SoftMax activation function, which is commonly used in binary classification tasks. The model is trained by employing the binary cross-entropy loss function in conjunction with the Adam optimizer.

#### 3.4.3. Third model (MobileNetV2 + Efficientnetv2_b0)

**Fig 6:**
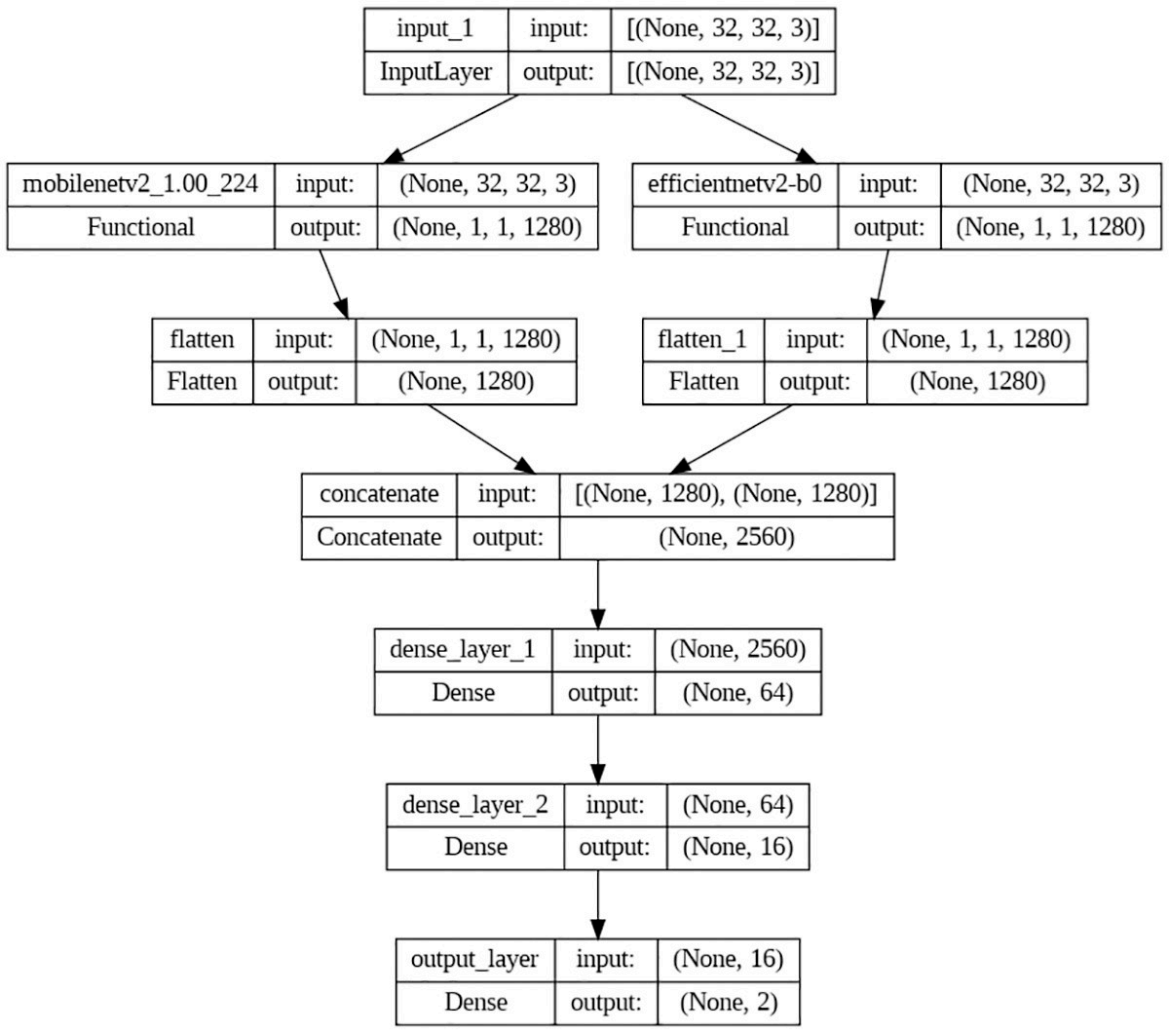
The architecture of the third model.

The (MobileNetV2 + EfficientNetV2_b0) model is a hybrid model that combines two pre-trained models, MobileNetV2 [32] and EfficientNetV2_b0 [33], to improve the accuracy of breast cancer classification. The MobileNetV2 model consists of 53 layers, including depthwise separable convolution layers, which reduce the computation required to perform convolutional operations without sacrificing accuracy. It also includes inverted residual blocks, which use skip connections to help the model learn more complex features. The EfficientNetV2_b0 model, on the other hand, is a highly efficient and scalable model that uses a compound scaling method to improve its performance. It has 24 layers, including multiple convolutional layers, and an attention mechanism that allows it to focus on the essential features of the input image. The input of both branches is also resized from 50 x 50 pixels to 32 x 32 pixels. The model configuration is shown below in Fig 6, with trainable parameters of 8,342,274.

In the hybrid model, the input image is first passed through the MobileNetV2 model, which extracts low-level features, followed by the EfficientNetV2_b0 model, which extracts high-level features. The output from both models is concatenated and fed into two dense layers, which perform binary classification using the Softmax activation function. Using two pre-trained models with different architectures allows the hybrid model to capture a broader range of features and improve the accuracy of breast cancer classification.

In our study, we employed the Adam optimizer with adaptive learning rate and decay to enhance the training process of our deep learning model. The Adam optimizer is well-known for its effectiveness in handling non-stationary and sparse gradients, making it suitable for a wide range of tasks, including image classification. By utilizing adaptive learning rates, the optimizer dynamically adjusts the learning rate for each parameter during training, which helps improve convergence and accelerates the learning process. Additionally, we incorporated learning rate decay, gradually reducing the learning rate over time. This strategy enables the model to fine-tune its weights and biases more effectively as it approaches convergence, preventing overshooting and instability during the later stages of training.

To further enhance the generalization capabilities of our model and mitigate the risk of overfitting, we employed the technique of early stopping. Overfitting occurs when a model becomes overly complex and memorizes the training data instead of learning generalizable patterns. The practice of early stopping entails the continuous evaluation of the model’s performance on a validation dataset during the training process. The training process is terminated prematurely in order to mitigate the issue of overfitting when the model’s performance on the validation set demonstrates a decline or reaches a point of plateaus. By selecting the model with the best validation performance, we ensure that our final model has learned meaningful representations from the data and is less likely to be affected by noise or irrelevant patterns. This combination of adaptive learning rate with decay and early stopping contributes to the robustness and effectiveness of our deep learning model for breast cancer classification tasks.

## 4. Experimental results and analysis

The execution of the models was carried out in Python, utilizing the TensorFlow and Keras libraries, which provided high-level tools for various layers. The three models were evaluated on the BC-IDC dataset, and the best-performing one was deemed our proposed model; this was then compared to other models previously established in this field. This experiment was performed on Google Colab, a product of Google Research, which was utilized to conduct these experiments. It enables individuals to write and run Python code in a browser environment. Using Colab is free, and the experiment setup utilized a Tesla K80 GPU and 12 GB of RAM, allowing for efficient and time-saving model building.

### 4.1. Performance metrics

The following model evaluates metrics: accuracy (ACC), precision (P), recall (R), F1-score, Matthew’s correlation coefficient (MCC), ROC-AUC, and PRC:

$$ACC=\frac{TP+TN}{TP+TN+FP+FN}$$ (3)

$$P=\frac{TP}{TP+FP}$$ (4)

$$R=\frac{TP}{TP+FN}$$ (5)

$$F1-score=2\times \frac{Precision\times Recall}{Precision+Recall}$$ (6)

$$MCC=\frac{TP\;x\;TN-FP\;x\;FN}{\sqrt{\left(TP+FP)(TP+FN)(TN+FP)(TN+FN\right)}}$$ (7)

Where True Positives=TP, False Positives=FP, False Negatives=FN, and True Negatives =TN.

### 4.2. Results on BC-IDC dataset

This section presents an analysis of the outcomes obtained from the examination of the three hybrid models that were offered.

#### 4.2.1. Performance analysis (ACC, Pres, Rec, MCC, ROC-AUC and PRC)

**Fig 7:**
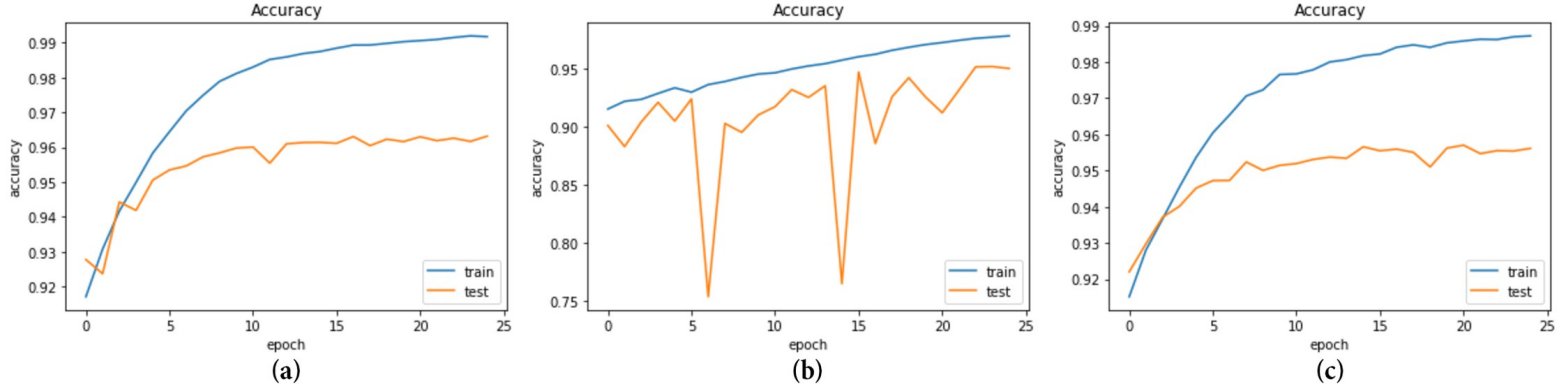
Accuracy of the three models during 25 epochs (a) CNN+EfficientNetV2B3; (b) MobileNet+Densenet121; (c) Mo-bileNetV2+Efficientnetv2_b0.

**Fig 8:**
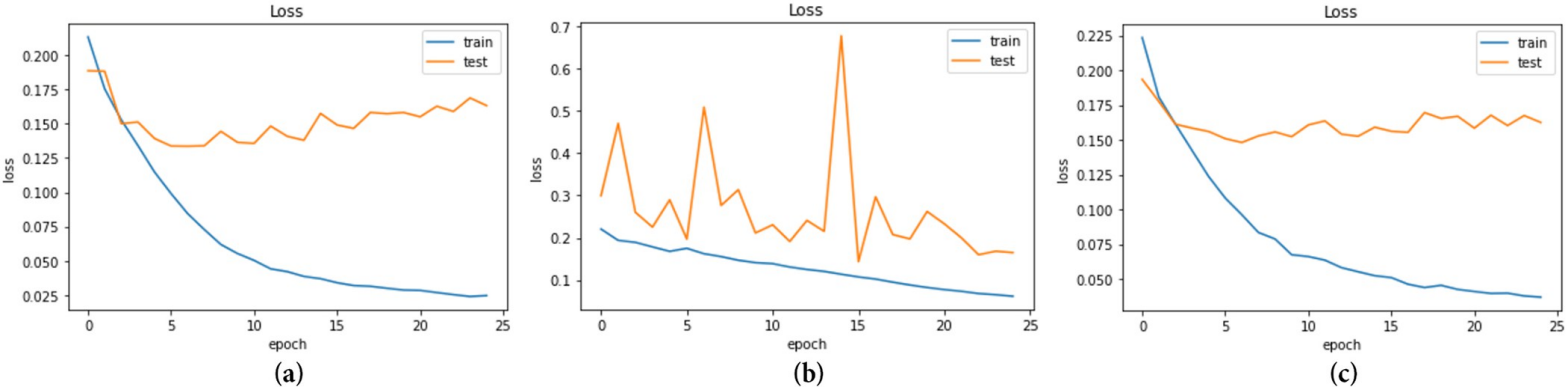
Loss of the three models during 25 epochs (a) CNN+EfficientNetV2B3; (b) MobileNet+Densenet121; (c) Mo-bileNetV2+Efficientnetv2_b0.

The models underwent 25 epochs of training, as depicted in Figs 7 and 8, which display the accuracy and loss curves, respectively.

Accuracy is a widely used performance measure on classification tasks such as cancer classification. The metric quantifies the ratio of accurate predictions generated by the model in relation to the overall number of predictions. The three models’ test accuracy is 96.3%, 95.1%, and 95.6%, respectively.

The training loss is a metric that indicates how close the model’s predictions are to the actual labels during training. It measures the discrepancy between the predicted labels and the real labels for the training dataset. The three models that attained test loss on non-trainable data are 0.163, 0.164, and 0.162, respectively.

To avoid over-fitting, we use an adaptive learning rate, which could be described as An enhancement of gradient descent techniques for the purpose of minimizing the objective function of a network. By using the gradient of the function and the parameters of the network.

We use an adaptive learning rate to keep the objective function of a network as low as possible without over-fitting, which could be thought of as an optimization of gradient descent methods. The objective function of the network is lowered by using the network’s parameters and the gradient of the function.

**Fig 9:**
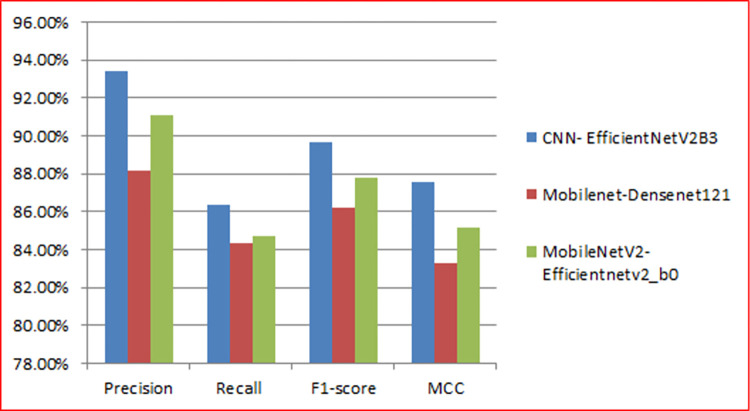
Precision, recall, F1-score and MCC of the three models during 25 epochs.

In Fig 9, precision, recall, F1-score, and Matthew’s correlation coefficient (MCC) are plotted to evaluate the performance of these models, respectively. These curves show how well the models perform regarding precision, recall, and MCC, which are important metrics in binary classification problems.

The proposed approaches achieved Matthews Correlation Coefficient (MCC) of 87.6%, 83.3%, and 85.2% with precision of 93.4%, 88.2%, and 91.1% with recall of 86.4%, 84.3%, and 84.7% with F1-score of 89.7%, 86.2%, and 87.8%, respectively.

**Fig 10:**
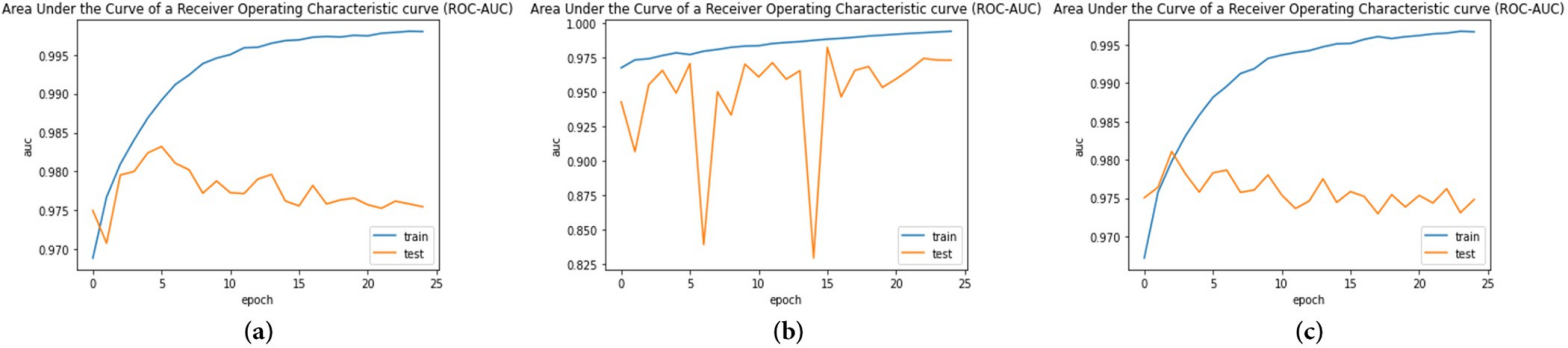
ROC-AUC of the three models during 25 epochs (a) CNN+EfficientNetV2B3; (b) MobileNet+Densenet121; (c) MobileNetV2+Efficientnetv2_b0.

**Fig 11:**
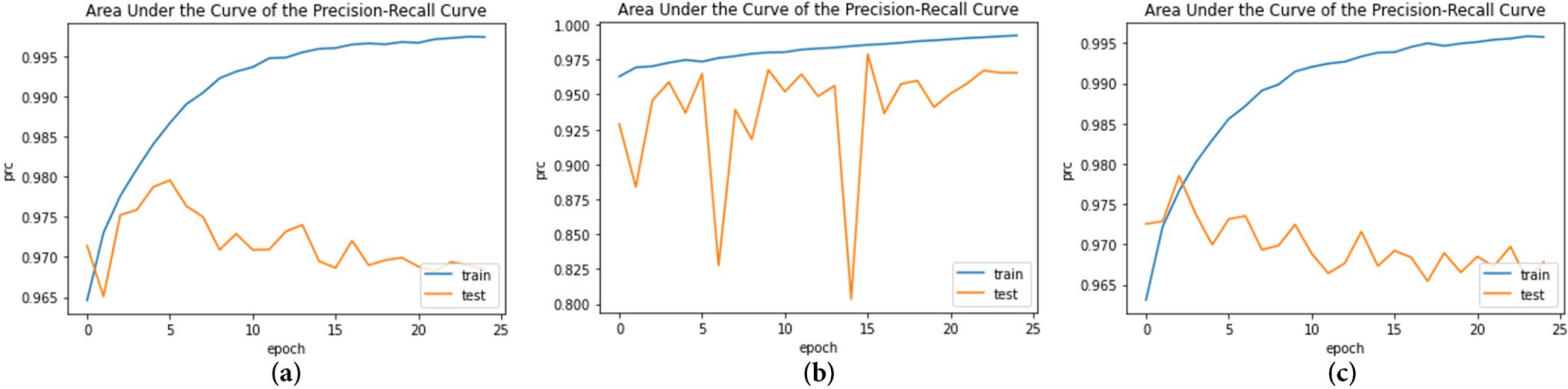
AUPRC of the three models during 25 epochs (a) CNN+EfficientNetV2B3;(b) MobileNet+Densenet121; (c) MobileNetV2+Efficientnetv2_b0.

The ROC-AUC and AUPRC curves were plotted after the models were trained over 25 epochs, as shown in Figs 10 and 11, respectively.

The ROC-AUC curve is created by plotting the true positive rate (TPR) against the false positive rate (FPR) at different classification thresholds. The three models attained test ROC-AUC of 97.5%, 97.2%, and 97.4%, respectively.

**Fig 12:**
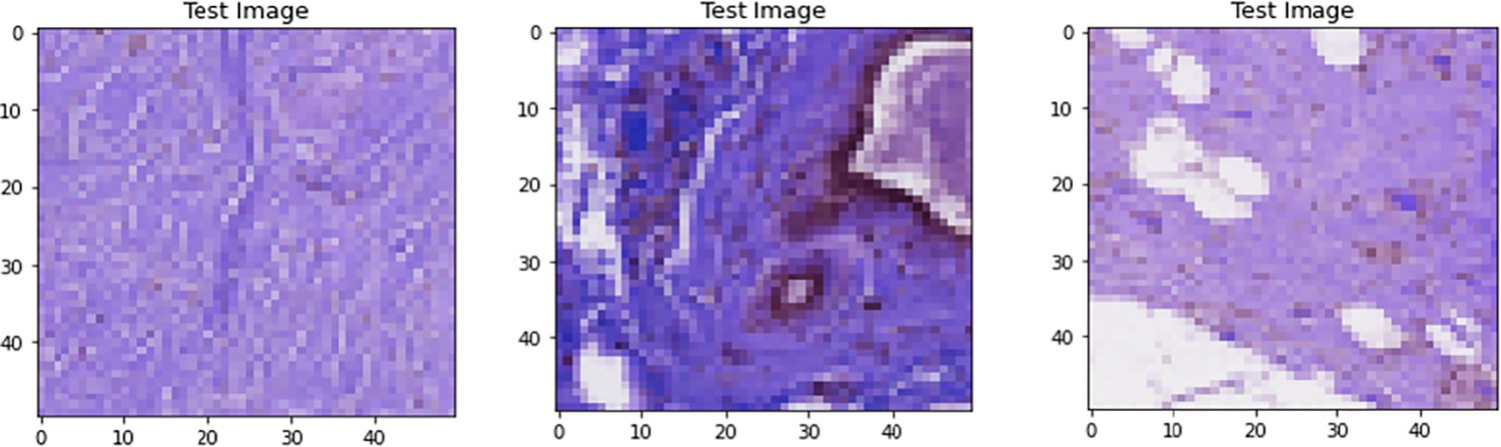
Predicted Value using three hybrid models (a) CNN+EfficientNetV2B3; (b) MobileNet+Densenet121; (c) MobileNetV2+Efficientnetv2_b0.

The AUPRC curve for the three models attained in the test dataset is 96.8%, 96.5%, and 96.7%, respectively. Fig 12 in the paper shows a collection of test images with their corresponding truth labels and the predicted class labels generated by the proposed models. The images demonstrate the effectiveness of the proposed models in correctly identifying the absence of breast cancer. For each test image, the predicted class label generated by the model is shown alongside the actual truth label. It is evident from the figure that the models have high accuracy in identifying the absence of breast cancer, as the predicted labels are the same as the actual labels for most of the test images. This high accuracy results from the models’ ability to capture the relevant features of the biopsy images through transfer learning from the pre-trained models and data preprocessing techniques to address data imbalances and enhance model performance.

#### 4.2.2. Confusion matrix

**Fig 13:**
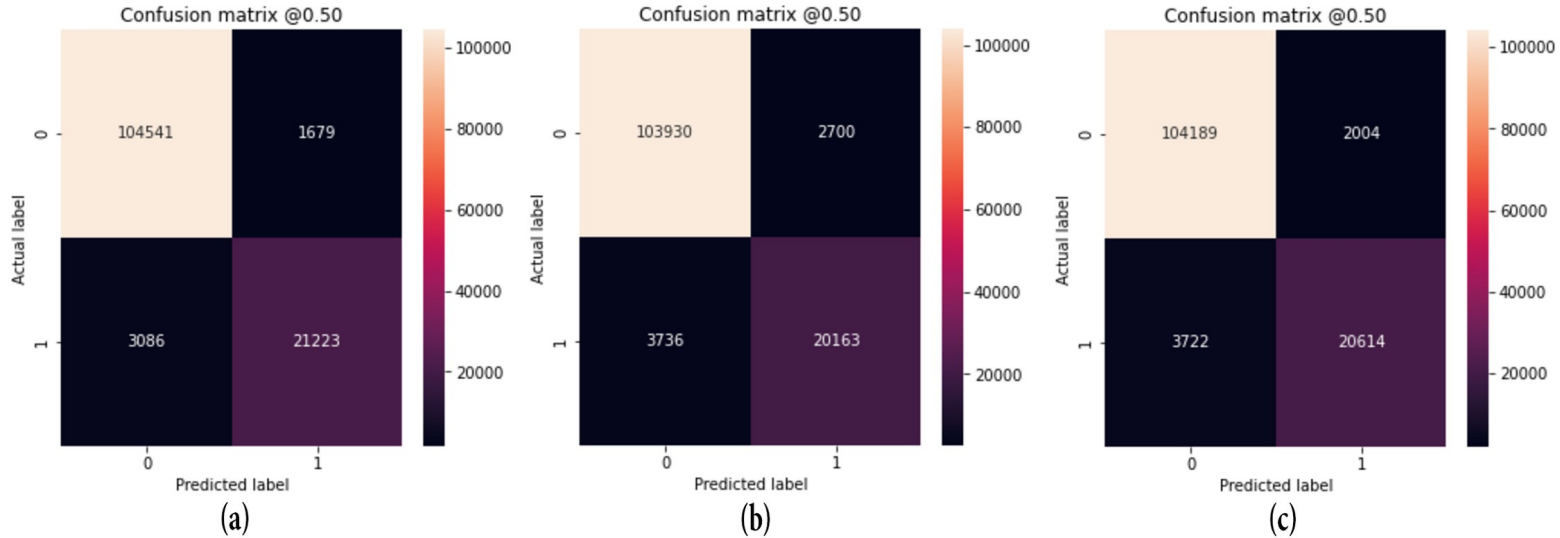
The confusion metric of the pre-trained models: (a) CNN+EfficientNetV2B3; (b) MobileNet+Densenet121; (c) MobileNetV2+Efficientnetv2_b0 for binary classification image.

A confusion matrix is a tabular representation utilized for assessing the effectiveness of a classification model. It summarizes the model’s actual and predicted classifications in a structured format. The confusion matrix typically The composition of the four values includes true positive (TP), false positive (FP), true negative (TN), and false negative (FN), as shown in Fig 13.

**Fig 14:**
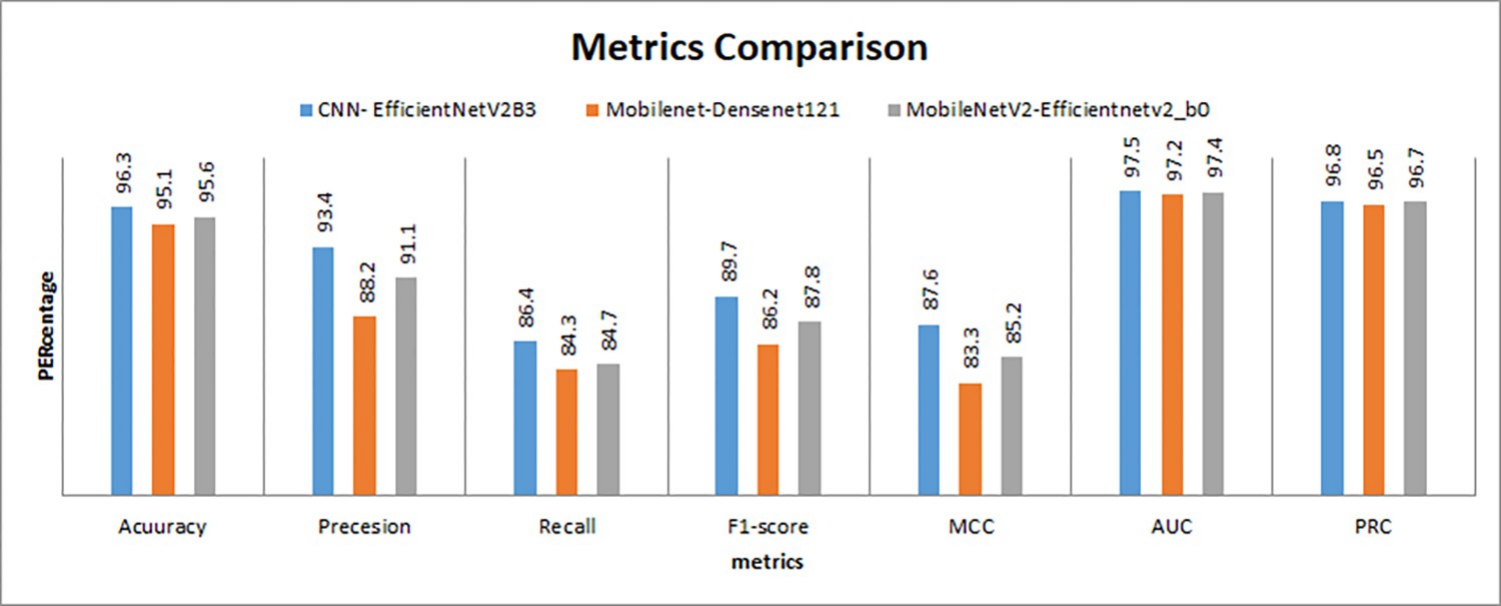
Metrics comparison (ACC, Pres, Rec, F1-score, MCC, ROC-AUC and AUPRC).

Then, the performance of the models through the various assessment metrics, such as accuracy, precision, recall, F1-score, MCC, ROC-AUC, and AUPRC) between the three hybrid models (CNN+EfficientNetV2B3, Mobilenet+Densenet121, and MobileNetV2+EfficientNetV2B0) has been made and is presented in Fig 14.

## 5. Comparison between the proposed models and models in the literature

Our proposed model, CNN+EfficientNetV2B3, has achieved a high accuracy of 96.3% on the BC-IDC dataset, which is significantly better compared to the other models mentioned above in the literature review section. Table 1 demonstrates the efficiency of the proposed model compared to the other models in the literature that have been applied to the same dataset; the table compares the test accuracy of the models.

**Table 1:** Comparison of the performance between the CNN+EfficientNetV2B3 method and other previous methods.

Author	Models	Accuracy
Barsha et al. [19]	DenseNet-121 and DenseNet-169	92.70%
Wang et al. [20]	CNN-GRU	86.21%
Choudhary et al. [21]	VGG19, ResNet34, and ResNet50	91.25%
Zeng et al. [22]	AutoML	91.6%
Celik et al. [23]	ResNet-50 and DenseNet-161	91.57%
Abdolahi et al. [24]	CNN	85%
Zhang et al. [25]	Alexnet, MobilenetV2, and Resnet50	87.45%
Sharmin et al. [27]	ResNet50V2 and ML	95%
**Proposed**	**CNN+EfficientNetV2B3**	**96.3%**

In the literature on classifying breast cancer into two distinct categories, researchers commonly rely on binary cross-entropy loss as the primary choice for the loss function in deep learning models. This loss function is designed specifically for binary classification tasks and effectively quantifies the disparity between predicted probabilities and the true ground truth labels. It has shown promising results and has been widely adopted due to its suitability for binary classification scenarios.

Moreover, it is noteworthy that the comparison among various studies in the literature utilizes the histopathology dataset and employs the same loss formula, i.e., binary cross-entropy. This standardized approach enables researchers to make fair and meaningful comparisons. Consequently, this contributes to establishing a reliable benchmark for evaluating and advancing breast cancer classification models effectively.

## 6. Discussion

In section 4, we saw that the accuracy of the first suggested approach, a combination of CNN and EfficientNetV2B3, on the histopathology images was higher than that of the second, a combination of MobileNet and DenseNet121, and the third, a combination of MobileNetV2 and EfficientNetV2_b0. Thus, we established the first approach as our own, making it a more stable and effective deep learning strategy.

The research, which integrates multiple pre-trained models, has displayed a relatively efficient runtime for processing. Time complexity is largely dependent on the operations performed, primarily the model size and depth, as well as the amount of input data. The quantity of layers and parameters in the model directly affects the space complexity, which represents the memory and storage needs of the model. The overall time consumption for each model is approximately 3 hours, such that the training time is approximately 2 hours, with almost 12 GB of memory usage.

The results achieved in this study are profoundly impactful, with the CNN+EfficientNetV2B3 hybrid model exhibiting an exceptional accuracy of 96.3% for breast cancer classification. This model outperforms existing methodologies in the literature, consistently showcasing accuracy, ROC-AUC, and AUPRC scores exceeding 95%. By combining the strengths of a convolutional neural network (CNN) with the efficient features extracted from the EfficientNetV2B3, trained on ImageNet, our hybrid approach successfully leverages both local and high-level semantic information, leading to its superior classification performance. These findings not only contribute academically but also hold immense practical implications. The precision in breast cancer detection provided by this model can substantially hasten the diagnostic process, potentially improving treatment outcomes and, consequently, patient survival rates. Future research directions could explore further enhancements, including the integration of additional image features, to continue advancing the field of medical image analysis.

### 6.1. The advantages and limitations of the proposed method

Despite the advantages of the first method in classifying breast cancer into IDC (+) or IDC (-), the first suggested method has several significant limitations that should be considered. The advantages and limitations include the following:

**At first the advantages:**

- By combining several powerful models, the suggested first approach attained the maximum accuracy by using a hybrid model that includes a CNN with the EfficientNetV2B3 model to extract features from histopathology images.
- EfficientNetV2B3 was trained on a large dataset (ImageNet). Researchers and practitioners may use pre-trained model weights and information gained from ImageNet.
- The performance of the first model was further improved by using the EfficientNetV2B3 model, which is one of the pre-trained models. High-level representations and semantic information were extracted from the input images using the EfficientNetV2B3 model, which operated as a feature extractor. The suggested model used transfer learning and eliminated the requirement to train the whole model from the start by using the EfficientNetV2B3 pre-trained weights. The result was increased speed and accuracy.
- The performance of the suggested techniques was assessed using comprehensive assessment measures such as accuracy, precision, recall, F1-score, MCC, AUC, and AUPRC. The authors comprehensively examined the model’s performance by including many assessment criteria. Through the first approach, we were able to establish the model’s superiority in obtaining high accuracy when compared to earlier approaches used for breast cancer diagnosis.

**Secondly, the limitations**

- CNN-EfficientNetV2B3 was only implemented for the classification of histological images. The findings presented in this study apply to a particular dataset and should not be generalized to other datasets.
- The histology images consist of 277,524 images that are 50x50 pixels each; thus, processing them will take a long time and use up a lot of memory in the collab to process the histology images; they are downsized to 32x32 pixels.
- The suggested model can aid effectively in classifying breast cancer histology images. Still, it is necessary to highlight that the pathologist should validate the final diagnosis because of the significance of the patient’s health and life.
- The scale of the image may affect its ability to extract certain patterns from it. In the proposed model, the image was resized to 32x32 pixels. As a result, some patterns may not be extracted, thereby diminishing the effectiveness of the proposed model. However, the proposed model outperformed the other models for the dataset under consideration.

**Future work**

- In future work, the potential for further improvement in the accuracy and robustness of the model can be explored by incorporating additional image features, such as texture features, including clinical data and patient history information, to provide a more comprehensive analysis, thereby refining the model’s predictions and aiding in better-informed decision-making.
- Handling Variations in Image Sizes: Investigate methods to handle variations in image sizes effectively, allowing the model to process diverse image resolutions and aspect ratios without losing critical information.
- Fine-Tuning and Transfer Learning: Experiment with different transfer learning techniques, fine-tuning strategies, and optimization algorithms to improve the model’s efficiency and adaptability to varied datasets.

## 7. Conclusion

In conclusion, this study highlights the novel CNN+EfficientNetV2B3 model, achieving an outstanding 96.3% accuracy by unifying multiple robust models. This approach demonstrated remarkable performance by integrating a hybrid model, combining a novel combination of custom CNN with EfficientNetV2B3 for feature extraction from histopathology images. The utilization of EfficientNetV2B3, trained on a large ImageNet dataset, offers a valuable resource for researchers and practitioners, allowing for the use of pre-trained model weights and insights obtained from ImageNet. Leveraging the EfficientNetV2B3 model as a feature extractor greatly enhanced the first model’s performance, yielding higher-level representations and semantic information from the input images. This approach capitalizes on transfer learning, obviating the need to train the entire model from scratch, thereby significantly boosting speed and accuracy. Robustness was extensively evaluated using comprehensive assessment measures such as accuracy, precision, recall, F1-score, MCC, AUC, and AUPRC. This study conclusively demonstrated the model’s superiority, achieving significantly higher accuracy compared to preceding methods employed in breast cancer diagnosis.

The successful amalgamation of diverse and powerful models significantly elevated the model’s overall performance, offering a multifaceted tool for breast cancer diagnosis. The incorporation of EfficientNetV2B3 brought a new dimension, ensuring not just accuracy but also speed and efficiency. By establishing robust performance across multiple assessment criteria, this research provides a strong foundation for enhancing breast cancer diagnosis tools. It reinforces the potential for future applications in medical image analysis.

## Abbreviations

CNN: Convolutional Neural Networks
IDC: Invasive Ductal Carcinoma
WSI: Whole Slide Images
BC: Breast Cancer
MCC: Matthew’s Correlation Coefficient
ROC-AUC: The Area Under the Curve of a Receiver Operating Characteristic
AUPRC: The Area Under the Curve of the Precision-Recall Curve
CAD: Computer Aided Detection
FDA: Food and Drug Administration
AI: Artificial Intelligent
ML: Machine Learning
DL: Deep Learning
TTA: Test Time Augmentation
GRU: Gated Recurrent Unit
MSRCNN: Multi-Scale Residual Convolutional Neural Network
